# Antibacterial activity of *Arthrobacter* strains isolated from Great Gobi A Strictly Protected Area, Mongolia

**DOI:** 10.3934/microbiol.2024009

**Published:** 2024-02-28

**Authors:** Alberto Bernacchi, Giulia Semenzato, Manuel di Mascolo, Sara Amata, Angela Bechini, Fabiola Berti, Carmela Calonico, Valentina Catania, Giovanni Emiliani, Antonia Esposito, Claudia Greco, Stefano Mocali, Nadia Mucci, Anna Padula, Antonio Palumbo Piccionello, Battogtokh Nasanbat, Gantulga Davaakhuu, Munkhtsetseg Bazarragchaa, Francesco Riga, Claudio Augugliaro, Anna Maria Puglia, Marco Zaccaroni, Fani Renato

**Affiliations:** 1 Department of Biology, University of Florence, Via Madonna del Piano 6, Sesto Fiorentino, 50019 Florence, Italy; 2 Department of Biological, Chemical and Pharmaceutical Sciences and Technologies-STEBICEF, University of Palermo, Viale delle Scienze Ed.17, 90128, Palermo, Italy; 3 Department of Health Sciences, University of Florence, viale G.B. Morgagni, 48, 50134 Firenze, Italy; 4 Department of Earth and Sea Science (DiSTeM), University of Palermo, Viale delle Scienze Blg. 16, Palermo, 90128, Italy; 5 Institute for Sustainable Plant Protection (IPSP)—National Research Council (CNR), Via Madonna del Piano 10, Sesto Fiorentino 50019 Florence, Italy; 6 Council for Agricultural and Economics Research (CREA) – Agriculture and Environment, Via di Lanciola 12/A, Cascine del Riccio, 50125, Florence, Italy; 7 Unit for Conservation Genetics (BIO-CGE), Institute for Environmental Protection and Research, via Ca' Fornacetta, 9, 40064 Ozzano dell'Emilia Bologna, Italy; 8 Consorzio Italbiotec, Piazza della Trivulziana 4/a 20126 Milano; 9 Institute of Biology, Mongolian Academy of Sciences, Peace Avenue-54B, Bayanzurkh District, Ulaanbaatar-13330, Mongolia; 10 Mongolian National University of Medical Sciences, S.Zorig street, Ulaanbaatar-14210, Mongolia; 11 Italian Institute for Envioronmental Protection and Research, via Vitalino Brancati, 48, 00144, Roma, Italy; 12 Wildlife Initiative, Bayangol, 6th Khoroo, Micro District 10, Ulaanbaatar, 210349, Mongolia

**Keywords:** Gobi Desert, *Arthrobacter*, inhibitory action, antibiotic resistance, VOCs

## Abstract

Desert soil hosts many microorganisms, whose activities are essential from an ecological viewpoint. Moreover, they are of great anthropic interest. The knowledge of extreme environments microbiomes may be beneficial for agriculture, technology, and human health. In this study, 11 *Arthrobacter* strains from topsoil samples collected from the Great Gobi A Strictly Protected Area in the Gobi Desert, were characterized by a combination of different techniques. The phylogenetic analysis, performed using their 16S rDNA sequences and the most similar *Arthrobacter* sequences found in databases, revealed that most of them were close to *A. crystallopoietes*, while others joined a sister group to the clade formed by *A. humicola, A. pascens*, and *A. oryzae*. The resistance of each strain to different antibiotics, heavy-metals, and NaCl was also tested as well as the inhibitory potential against human pathogens (i.e., *Burkholderia* ssp., *Klebsiella pneumoniae, Pseudomonas aeruginosa*, and *Staphylococcus* ssp.) via cross-streaking, to check the production of metabolites with antimicrobial activity. Data obtained revealed that all strains were resistant to heavy metals and were able to strongly interfere with the growth of many of the human pathogens tested. The volatile organic compounds (VOCs) profile of the 11 *Arthrobacter* strains was also analyzed. A total of 16 different metabolites were found, some of which were already known for having an inhibitory action against different Gram-positive and Gram-negative bacteria. Isolate MS-3A13, producing the highest quantity of VOCs, is the most efficient against *Burkholderia cepacia* complex (Bcc), *K. pneumoniae*, and coagulase-negative Staphylococci (CoNS) strains. This work highlights the importance of understanding microbial populations' phenotypical characteristics and dynamics in extreme environments to uncover the antimicrobial potential of new species and strains.

## 1. Introduction

Desert ecosystems represent a considerable portion (more than 30%) of the global land area [1]. The main features of these ecosystems are the near-complete absence of precipitation (<250 mm average annual precipitation) and high evapotranspiration as the result of the combination of different climatic factors [2],[3]. In desert regions, soil aridity is influenced by different factors, such as wind erosion, temperature fluctuations, and sedimentation. Moreover, most desert soils lack many organic compounds and nitrogen, while being rich in salt ions, phosphate, magnesium, and calcium carbonates [4]. All these abiotic factors have a significant impact on soil biome [2].

Microorganisms are indispensable components in soils since they govern the biogeochemical cycling of macronutrients and micronutrients vital for plants and animal life. Microbial diversity and activity are apparently limited in desert ecosystems. However, many studies suggest a considerable presence of microorganisms, although to a lesser extent than in temperate soils, and the presence of microbial consortia that provide robustness and extensive metabolic capabilities, thus enabling them to establish important relationships with both eukaryotic and prokaryotic organisms in desert environments [5]. Moreover, many desert areas are characterized by the presence of biological soil crusts (BSCs) formed by soil particles mixed with both eukaryotic and prokaryotic microorganisms constituting the soil surface microbiological communities [6]. The BSCs can improve soil fertility by capturing the essential nutrient-rich dust and by holding water, thus stabilizing and nourishing the soil [4]. The bacterial communities composing the BSCs can help in fixating nitrogen and carbon, synthesizing organic compounds, and releasing them in the surrounding environment, thus contributing to the increment in microbial biomass and plant growth [6]. A better understanding of desert soil microbial communities and the extensive characterization of individual species with plant-growth promoting activities might lead to the exploitation of such microbes in sustainable agriculture practices [4], in the prevention of desertification processes, and to restore vegetation cover [7].

Furthermore, from an evolutionary perspective, understanding how bacterial communities have adapted and evolved in these extreme environments can provide valuable insights into their resilience and survival and could help predict how bacterial communities might respond to future environmental changes. From an ecological viewpoint, examining how these bacterial communities interact within extreme ecosystems can reveal important dynamics of nutrients and energy supply with important implications for the conservation and management of ecosystems. Finally, the search for thermostable and alkaline-stable enzymes and new molecules with antibiotic properties is crucial for biotechnological and pharmaceutical applications [8].

The Great Gobi A Strictly Protected Area is one of the wild areas of the Trans-Altai Gobi Desert that remained almost intact, covering 44,190 km^2^ of uninhabited dryland. This area is characterized by extreme temperatures and variable precipitation levels across southern Mongolia and northwestern China. The extremely harsh environment reflects a unique ecosystem that provides a critical habitat for various rare species of flora and fauna [9].

Since it is a strictly protected area with extreme weather conditions, the Great Gobi A represents an intriguing model to study bacterial communities. Most studies on the Great Gobi environment and ecology are focused on plant growth and animal diversity, while analyses of the bacterial communities are scarce and limited to independent cultivable approaches [10]. This study aimed to characterize, by a combination of molecular and phenotypic tests, cultivable bacteria isolated from soil samples of two different oases of Great Gobi A, focusing on 11 *Arthrobacter* isolates to check their ability to interfere with the growth of human pathogens through the synthesis of bioactive molecules. This idea relies on the finding that bacteria inhabiting extreme environments (i.e., Antarctica) can strongly interfere with the growth of human pathogens, such as those belonging to the *Burkholderia cepacia* complex (Bcc) [11]–[13], and that the inhibitory activity shown by these bacteria might rely also on the biosynthesis of volatile organic compounds (VOCs) [14],[15]. It is thought that the production of antimicrobial agents may help bacteria living in extreme environments in the competition for scarce resources by keeping the competitor populations low [16]. Moreover, it has been highlighted that many environmental bacteria show antibiotic resistance and that the multiple antibiotic resistance (MAR) operon and other resistance-related genes are maintained, suggesting the presence of selective pressures that allow the acquisition and retention of such genes [16]. This phenomenon may not only depend on the continuous exposure to natural antibiotics since many of the resistance-related mechanisms are also used for the detoxification of the cell environment from heavy metals and toxins [16]. Another aspect is that sub-inhibitory concentrations of antibiotics play an important role in the regulation of gene expression and in cell-to-cell communication, allowing for biofilm formation and population control [17]. Thus, analysing the inhibitory potential of bacteria isolated from the Gobi Desert may improve our knowledge regarding natural antimicrobial organic compounds and the means of communication of complex bacterial communities.

## 2. Materials and methods

### 2.1. Soil sampling and cultivable bacteria isolation

Five topsoil samples were collected from each of two oases in the Great Gobi A Strictly Protected Area (Oasis 2: Lat. 43,35308333; Long. 96,34411661 and Oasis 3: Lat. 43,30285; Long. 97,77906667). The samples are marked by the number of the correspondent oasis and with a letter (ranging from A to E). The cultivable bacteria were isolated from each soil sample. To accomplish this, 20 mL of NaCl 0.9%, 10 g of glass beads, and 2 g of soil sample were mixed and incubated at room temperature under horizontal shaking (250 rpm) for 1 hour. After a centrifugation at 1000 rpm for 2 minutes, the supernatant was collected and diluted (10^−1^ and 10^−2^). The dilutions were plated on tryptic soy agar (TSA) medium and incubated for 48 hours at 25 °C. For each dilution, the vital titer was evaluated. Twenty-five colonies from each sample were randomly selected and isolated onto new TSA plates. Each isolate was then stored at −80 °C in 20% glycerol.

### 2.2. Bacterial strains and growth conditions

The 11 *Arthrobacter* strains will be referred to as MS-2A2, MS-2A14, MS-3A3, MS-3A4, MS-3A8, MS-3A11, MS-3A13, MS-3A17, MS-3A20, MS-3A21, and MS-3A25, where “M” stands for “Mongolia”, “S” for “soil”, the first number identifies the oasis, the letter the sample, and the last number refers to the isolate. The *Arthrobacter* isolates were grown on TSA plates at 25 °C for 48 h.

The bacterial strains used as targets (Table 1) in the cross-streaking test (see paragraph 2.8) are Bcc strains, grown on LB (Luria-Bertani) plates at 37 °C for 48 h, and a panel of 36 strains of *Staphylococcus aureus*, *Staphylococcus haemolyticus*, *Staphylococcus epidermidis*, *P. aeruginosa*, and *K. pneumoniae*, which were grown on TSA medium at 37 °C for 24 h. The resistance to antibiotics of these pathogenic strains has been previously characterized by the disk-diffusion method [18]. *K. pneumoniae* ATCC 700603, *P. aeruginosa* ATCC 27853, and *S. aureus* ATCC 25923 were obtained from Thermo Fisher Diagnostics S.p.A. The other *S. aureus*, CoNS, *P. aeruginosa*, and *K. pneumoniae* strains were isolated and provided by the applied microbiology laboratory in the Health Sciences Department of the University of Florence, Italy.

### 2.3. Sequencing of 16S rDNA coding genes

The 16S rDNA coding genes from bacterial isolates were amplified according to the protocol described by Semenzato et al. (2023) [19], with slight modifications. In a final volume of 20 µL, 1 x Xpert TaqBuffer, 2 mM MgCl_2_, 0.20 mM dNTPs, 0.6 µM primers P0 (5′ GAGAGTTTGATCCTGGCTAG 3′) and P6 (5′ CTACGGCTACCTTGTTACGA 3′) ([19]), 1 U of Dream Taq DNA Polymerase (Thermo Scientific) and 1 µL of thermal lysates were mixed. The DNA fragments were amplified through 30 thermal cycles with the following profile: 95 °C for 30 s, 50 °C for 30 s, 72 °C for 1 minute. A final extension at 72 °C for 10 minutes was then applied. Amplicons were analyzed through a 0.8% agarose gel electrophoresis and purified with the A'SAP PCR clean-up kit (ArcticZymes Technologies ASA, Tromsø, Norway). Amplicon sequencing was performed using the BigDyeTM Terminator Cycle Sequencing chemistry (AppliedBiosystems, Foster City, CA, USA), and the sequences were visualized through the software Chromas 2.6.6 (Technolysium Pty Ltd). Sequences were then compared with those available in the NCBI database for the taxonomic affiliation of bacteria isolates. The 16S rDNA sequences from 11 isolates obtained from samples 2A and 3A were submitted to GenBank and were assigned the accession numbers OR712066–OR712076.

### 2.4. Random amplified polymorphic DNA (RAPD) analysis

The RAPD analysis was performed on thermal lysates from the 11 *Arthrobacter* isolates. Thermal lysates were obtained by resuspending single colonies in 20 µL of sterile purified water, incubating the solution at 95 °C for 10 minutes, and then leaving it in ice for 5 minutes [19]. Lysates were then centrifuged at 13,000 rpm for 3 minutes to separate the DNA-containing supernatant from the cellular pellet. 2 µL of each lysate were then used to perform an amplification reaction with the following reaction mix, according to [19]: DreamTaq DNA polymerase (Thermo Fisher Scientific) 1 U, dNTPs 0.20 mM, DreamTaq Buffer 1X, 500 ng of 1253 primer (5′ GTTTCCGCCC 3′,[20]). After an incubation of 1 minute at 90 °C and 95 seconds at 95 °C in a thermal cycler, the reaction mix was subjected to 45 cycles of 95 °C for 30 seconds, 36 °C for 1 minute, and 75 °C for 2 minutes. The reaction mixtures were incubated for 10 minutes at 75 °C and 65 °C for additional 10 minutes. Amplicons were visualized through 2% w/v agarose gel electrophoresis and the RAPD profiles of each isolate were compared for the presence/absence of shared bands. Isolates sharing the same amplification pattern were assigned to the same haplotype (strain).

### 2.5. Phylogenetic analysis

The nucleotide sequences of the 16S rRNA coding gene obtained for the 11 *Arthrobacter* isolates were aligned with those of type strains retrieved from the Ribosomal Database Project using the muscle algorithm [21]. The sequence alignment was used to build a phylogenetic tree, using the program Mega XI [22]. The 16S rDNA sequences of both type strains and those belonging to the bacteria isolated in this work were reduced to the same length using the muscle parameter. The neighbor-joining algorithm was applied with a 1000-bootstrap resampling and the distance matrix was constructed using the Kimura two-parameters (K2P) model. These parameters were chosen since the analyzed microorganisms belong to the same genus and have closely related sequences.

**Table 1.**
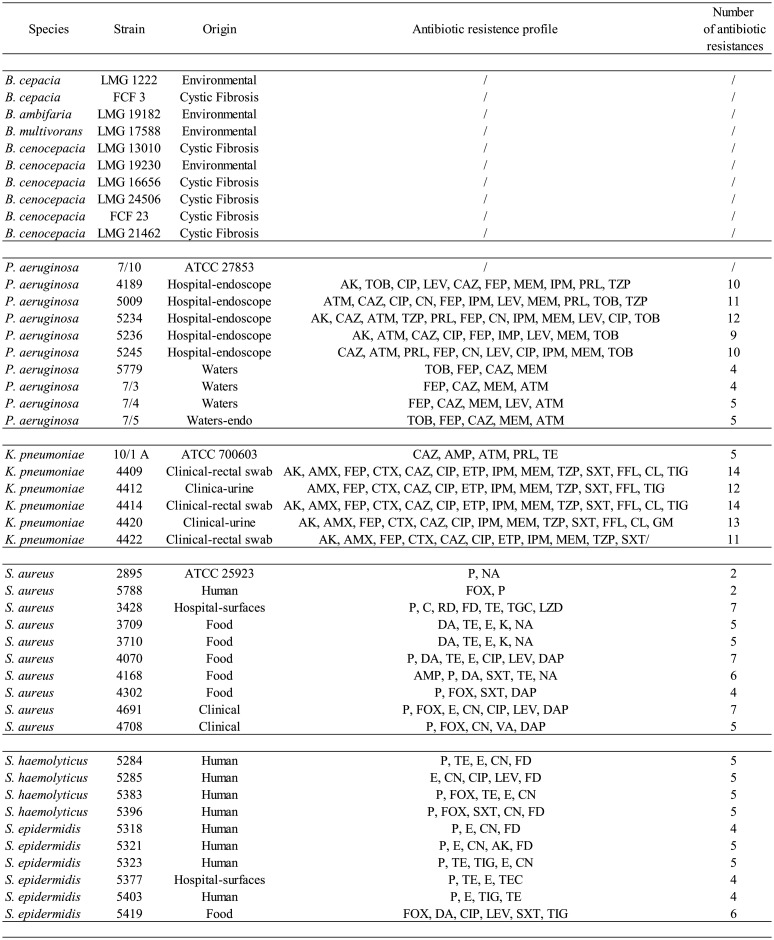
List of bacterial strains used in this work.

Abbreviations: AK: amikacin; AMX: amoxicillin; AMP: ampicillin; ATM: aztreonam; CTX: cefotaxime; CAZ: ceftazidime; CIP: ciprofloxacin; CN: gentamicin; DA: clindamycin; DAP: daptomycin; ETP: ertapenem; E: erythromycin; FD: fusidic acid; FEP: cefepime; FOX: cefoxitin; IPM: imipenem; K: kanamycin; LEV: levofloxacin; MEM: meropenem; NA: nalidixic acid; P: penicillin G; PRL: piperacillin; SXT: sulfamethoxazole/trimethoprim; TE: tetracycline; TEC: teicoplanin; TIG: tigecycline; TOB: tobramycin; TZP: piperacillin/tazobactam; VA: vancomycin.

### 2.6. Plasmid extraction

Plasmid DNA was extracted from each *Arthrobacter* strain using the QIAscreen protocol as follows: cells were inoculated in 5 mL of tryptic soy broth (TSB) for 24 hours at 25 °C under shaking (130 rpm). 3 mL of these cultures were centrifuged at a speed of 13.000 rpm for 3 minutes, the supernatant was discarded, and the pellet was resuspended in 0.3 mL of P1 solution (50 mM Tris-HCl Ph 8, 10 mM EDTA pH 8, RNase A 400 µg/mL). 0.3 mL of pre-heated (50 °C) P2 solution (200 mM NaOH, 1% SDS) were added to the suspension and the obtained solution was incubated for 5 minutes at room temperature. 0.3 mL of P3 solution (2.55M K-acetate pH4.8) were added and the obtained solution was centrifuged for 15 minutes at room temperature. 700–800 µL of the supernatant were recovered and the precipitation of the DNA was obtained by adding 8 volumes of isopropanol at room temperature. The solution was centrifuged for 15 minutes at room temperature and the pellet was washed twice with 700 µL of EtOH 70%. Plasmid DNA was resuspended in an appropriate volume of dH_2_O and visualized through agarose gel electrophoresis.

### 2.7. Growth of Arthrobacter isolates in the presence of antibiotic, heavy metals, and NaCl

The resistance of the 11 *Arthrobacter* isolates to different antibiotics, heavy metals, and sodium chloride was evaluated. The isolates were streaked on TSA medium containing different concentrations of each compound and their growth was checked after a period of incubation of 48 hours at 25 °C. The antibiotic and heavy metals used in this work were: streptomycin (0.5; 1; 2.5; 5; 10; 50 µg/mL); kanamycin (0.5; 1; 2.5; 5; 10; 50 µg/mL); ciprofloxacin (0.5; 1; 2.5; 5; 10; 50 µg/mL); tetracycline (0.5; 1; 2.5; 5; 10; 50 µg/mL); chloramphenicol (0.5; 1; 2.5; 5; 10; 50 µg/ mL); rifampicin (0.5; 1; 2.5; 5; 10; 50 µg/mL); Cu (CuCl_2_ 1; 2.5; 5; 10 mM); Ni (NiCl_2_ 1; 2.5; 5; 10 mM); Zn (ZnSO_4_ 1; 2.5; 5; 10 mM); As(III) (NaAsO_2_ 1; 2.5; 5; 10 mM); As(V) (KH_2_AsO_4_ 1; 2.5; 5; 10 mM). The resistance to different concentrations of NaCl was evaluated by streaking the isolates on TSA supplemented with 1%, 2%, 4%, 6%, 8%, and 10% of NaCl. For each experiment, the growth of each isolate was compared to their growth on a control plate (TSA medium), and the minimal inhibitory concentration (MIC) was obtained.

### 2.8. Cross-streaking

Cross-streaking tests were carried out as follows. Each tester *Arthrobacter* strain was plated on one half of a Petri dish containing TSA medium to test their inhibition potential against pathogens (targets). Tester strains were incubated at 25 °C for 48 hours. In the meantime, target strains (Table 1) were isolated on LB (Bcc strains) or TSA media (*Staphylococcuss* ssp., *P. aeruginosa*, and *K. pneumoniae* strains) for 24 hours at 37 °C. Bcc strains required 48 hours of incubation.

A few colonies of each strain were then diluted in 100 µL (Bcc strains), 35 µL (coagulase-negative strains (CoNS)) or 50 µL (all the other target strains) of saline solution (NaCl 0.9%) and streaked on the second half of the Petri dishes, perpendicularly to the middle line. Each Petri dish could host 10 different streaks. Growth controls were made by streaking the targets without any tester. Plates were incubated according to the necessities of each target strain. After the incubation period, the inhibitory activity of the testers against each one of the targets was evaluated by comparing the growth of each target with the growth control. The inhibitory activity was classified on a scale from 0 to 3 where 0 is “no inhibition” and 3 is “complete inhibition”.

The same protocol was adopted to test the production of VOCs with inhibitory action, using Petri dishes divided into two compartments by a physical septum.

### 2.9. HS-GC/MS analysis of VOCs

Biomass from bacterial culture (30–100mg) was collected in headspace (HS) vials. Vials were sealed and immediately analyzed. HS-vials were conditioned at 40 °C for 20 minutes before extraction. Bacterial VOCs produced by bacterial isolates were extracted from the vial headspace and injected in the gas chromatograph (GC). Headspace extraction was performed with a 2.5 mL Syringe-HS (0.64-57-R-H, PTFE, GERSTEL) conditioned and held at 40 °C from sample collection to injection. Splitless injection was used. Gas chromatographic analysis was performed adapting a previously reported method [23]. An Agilent 7000C GC (Agilent Technologies, Inc., Santa Clara, CA, USA) system equipped with a split/splitless injector, fitted with an Agilent HP5-MS UI capillary column (30 m × 250 µm; 0.25 µm film thickness), coupled to an Agilent triple quadrupole Mass Selective Detector MSD 5973 (Agilent Technologies, Inc., Santa Clara, CA, USA) was used; ionization voltage 70 eV; electron multiplier energy 2000 V; transfer line temperature, 270 °C; solvent delay: 0 min. Helium was used as the carrier gas (1 mL min^−1^). The oven program was as follows: the temperature was initially kept at 40 °C for 5 min. Then, the temperature was gradually increased to 250 °C at a 2 °C/min rate. The temperature was held for 15 min and finally raised to 270 °C at a 10 °C/min rate. Samples were injected at 250 °C automatically. Interval scan: 35–450 m/z; Scan speed: 10,000 amu·s^−1^ (25 Hz). The GC–MS mass spectrum data were analyzed using the MassHunter Qualitative Analysis B.06.00 and the National Institute Standard and Technology (NIST) database was used to interpret analyzed data. A comparison of the mass spectrum of the unidentified components released by the bacterial isolates was carried out against the mass spectrum of already known components available in the NIST 11 MS library.

## 3. Results

A panel of more than one hundred bacterial isolates (most of which belonging to different Gram positive genera, such as *Arthrobacter*, *Pseudoarthrobacter*, *Oceanobacillus*, *Bacillus*, and *Peribacillus*) obtained from the two oases was checked for their ability to inhibit the growth of two multidrug-resistant Bcc strains (i.e., FCF3 and LMG16656, belonging to *B. cepacia* and *B. cenocepacia* species, respectively, and isolated from Cystic Fibrosis patients) in order to identify the most efficient isolates in producing antibacterial compounds. Data obtained revealed that several isolates were able to strongly inhibit the growth of both CF strains (not shown), with eleven of them exhibiting the highest degree of inhibition. These eleven isolates were then chosen for further analyses.

### 3.1. Random amplified polymorphic DNA analysis of Arthrobacter isolates

To check whether the 11 isolates from Gobi Desert soil samples corresponded to the same or to different strains, an RAPD analysis was performed as described in Section 2. The 11 RAPD profiles (haplotypes) were then compared and, assuming that isolates sharing the same RAPD fingerprinting correspond to the same bacterial strain ([24] and references therein), 11 different RAPD haplotypes were identified, thus corresponding to 11 different strains (Supplementary Figure S1).

### 3.2. Taxonomy affiliation and phylogeny of the 11 Arthrobacter strains

**Figure 1.**
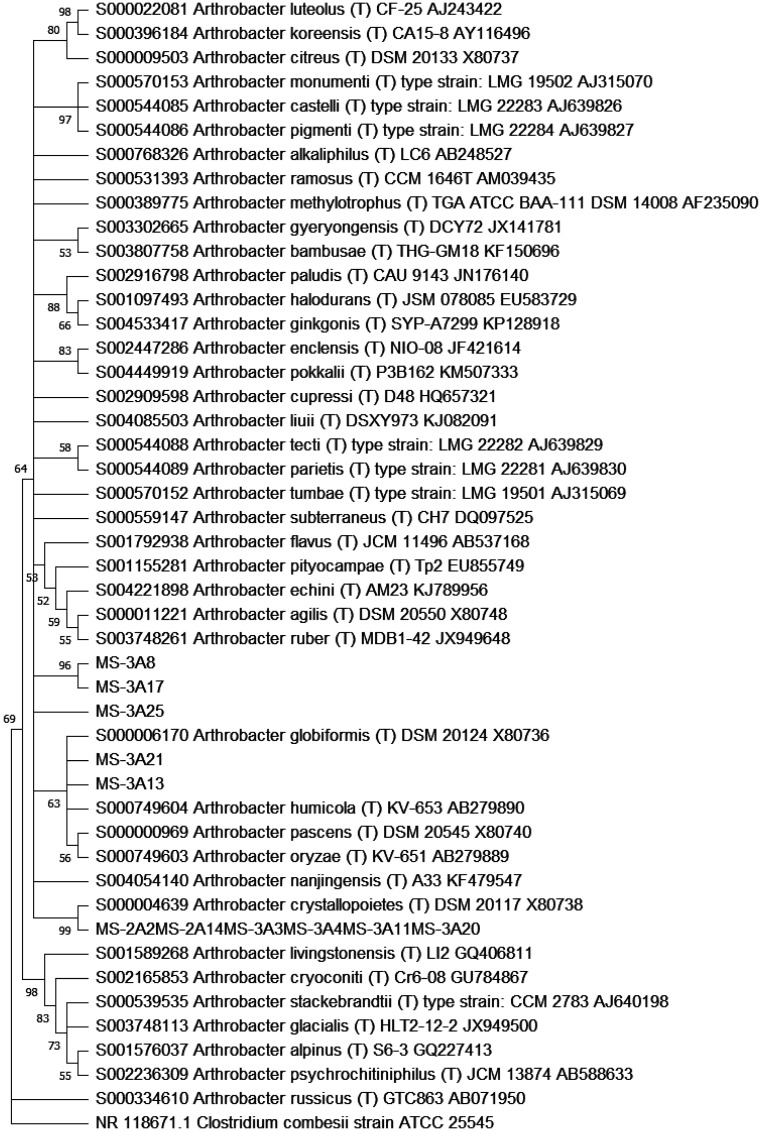
Phylogenetic tree of *Arthrobacter* strains based on 16S rRNA coding genes. The *Clostridium combesii* orthologous sequence was used as an outgroup.

The 16S rDNA sequence was amplified and sequenced from each of the 11 bacterial strains. The obtained sequences were then used to probe the databases by the BLASTn program. Each sequence retrieved orthologous sequences belonging to the genus *Arthrobacter* at the highest degree of sequence identity. The 11 sequences and the most similar ones were then used to build a phylogenetic tree as described in Section 2 (Figure 1). Strains with a 16S rDNA sequence identity higher than 97% were considered to belong to the same operational taxonomic unit (OTU). *Arthrobacter* sp. MS-2A2, MS-2A14, MS-3A3, MS-3A4, MS-3A11 and MS-3A20 shared the same 16S rDNA sequence, constituting a single OTU. They were phylogenetically very close to the species *Arthrobacter crystallopoietes*, while MS-3A21 was close to *Arthrobacter globiformis*. MS-3A13 joined a sister group to the clade formed by *Arthrobacter humicola, A. pascens* and *A. oryzae*, while MS-3A8, MS-3A17 and MS-3A25 were phylogenetically close and form a clade themselves, showing a degree of sequence identity of 99% between MS-3A8 and MS-3A17, 97% between MS-3A8 and MS-3A25, and 97% between MS-3A17 and MS-3A25.

### 3.3. Antibiotic, heavy metals, and NaCl resistance profiles

The resistance of the bacteria to different concentrations of antibiotics, heavy metals, and NaCl was evaluated, and the MIC was obtained for each compound.

Each *Arthrobacter* strain was able to resist the highest concentrations of kanamycin and streptomycin used, while being less tolerant towards chloramphenicol, ciprofloxacin, rifampicin, and tetracycline. The MIC value for tetracycline was <0.5 µg/mL for each strain. No growth was observed for any of the strains at the minimal concentration of chloramphenicol (MIC < 1 µg/mL) and rifampicin (MIC <5 µg/mL) used in this experiment. 54% of the isolates did not grow in the presence of 0.5 µg/mL ciprofloxacin, while 62% tolerate a concentration of >10 µg/mL of streptomycin. 100% of the *Arthrobacter* strains showed capacity to grow in the presence of the highest concentration of kanamycin used (MIC > 50 µg/mL). In order to check whether the antibiotic resistance exhibited by the *Arthrobacter* strains might be due to the presence of plasmids, the plasmid DNA was extracted from each strain as described in Section 2. Data obtained (not shown) revealed the absence of plasmid molecules. However, we cannot a priori completely rule out the possibility of the presence of plasmids larger than those detectable with the method used in this work.

The 11 *Arthrobacter* strains could grow in the presence of moderate concentrations of KH_2_AsO_4_ and NaAsO_2_, while their growth was inhibited at low concentrations of CuCl_2_, ZnSO_4_, Cd(NO_3_), and NiCl_2_. In particular, no growth was observed for any of the strains at the minimum concentration of ZnSO_4_ (MIC < 5 µg/mL), Cd(NO_3_) (MIC < 5 µg/mL), and NiCl_2_ (MIC < 5 µg/mL) used in this experiment. 62% of the strains were not able to grow in the presence of the minimal concentration of NaAsO_2_ that has been used for this test (0.5 µg/mL). 69% of the *Arthrobacter* strains tolerated a concentration of 10 µg/mL of KH_2_AsO and of 5 µg/mL of CuCl_2_. However, it is necessary to remark that, even if the percentage of strains resistant to the maximum concentration of KH_2_AsO and CuCl_2_ were the same, the resistant strains were different. The 11 *Arthrobacter* strains tolerate concentrations below 2% of NaCl. Concentrations higher than 4% lead to total growth inhibition for most of the strains. Data obtained are shown in Table 2.

To understand whether these characteristics might be shared by strains belonging to the *Arthrobacter* genus but isolated from different environmental niches, a comparison has been made between the MIC of different antibiotics and heavy metals exhibited by the “Mongolia Soil” strains and by a panel of *Arthrobacter* and *Pseudarthrobacter* strains associated with *Origanum heracleoticum* L. [25]. A phylogenetic tree (Supplementary Figure S4) has been made to understand the relationship between these strains. As shown in Supplementary Figure S5, the *Arthrobacter* strains isolated from Gobi Desert are generally less resistant to streptomycin, kanamycin and to arsenate (As(V)) induced stress. A principal component analysis (PCA) was performed, and the results remark the difference in the resistance profiles between bacteria belonging to different strains and isolated in different contexts (Supplementary Figure S6).

**Table 2.**
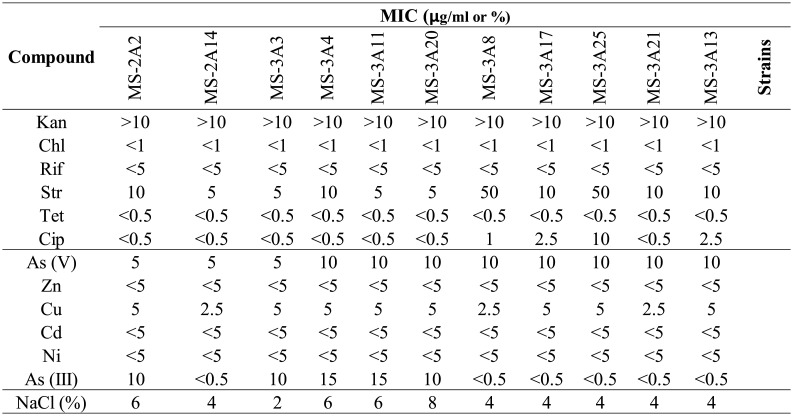
MIC of antibiotic kanamycin (Kan), chloramphenicol (Chl), rifampicin (Rif), streptomycin (Str), tetracycline (Tet), and ciprofloxacin (Cip); heavy metals (As(V), Zn, Cu, Cd, Ni, As(III) ); and NaCl. MICs were measured in µg/ml for antibiotics and heavy-metals and in percentage for NaCl.

### 3.4. Antimicrobial activity of Arthrobacter strains

The 11 *Arthrobacter* strains were tested against (Bcc), *S. aureus*, CoNS (*S. haemoliticus* and *S. epidermidis)*, *P. aeruginosa*, and *K. pneumoniae* strains, to check their ability to produce diffusible and/or volatile molecules with antibacterial properties. To this aim, the cross-streaking method was used as described in Section 2. We first checked the production of bioactive molecules using standard Petri dishes. We then checked the inhibitory activity of VOCs produced by *Arthrobacter* strains by using Petri dishes with a septum separating the two halves [23]. Data obtained are shown in Tables 3–5. For each test, an inhibition score (I.S.) was calculated by adding the inhibition values assigned to each tester against any target and normalized by the number of targets (10 Bcc strains, 10 CoNS strains, 6 *K. pneumoniae* strains, and 10 *P. aeruginosa* strains; Table 3).

Data obtained using the Bcc strains (Supplementary Figures S2 and S3) as targets showed that, in both cases (i.e., Petri dishes with or without septum), all the *Arthrobacter* strains were able to inhibit the growth of Bcc strains. This finding demonstrates that all the 11 *Arthrobacter* strains were able to synthetize diffusible and/or volatile organic compounds with a (strong) anti-Bcc activity. The *Arthrobacter* isolates with the highest I.S. against Bcc in both conditions (with and without septum) were MS-3A13, MS-3A4, MS-2A2, MS-A325, and MS-3A20. The sensitivity score (S.S.) for each Bcc strain was calculated by adding the inhibition values for each tester and normalized by the number of testers (11 *Arthrobacter* strains). In both conditions (i.e., with and without septum), the most sensitive Bcc strains were FCF3 (S.S. = 2.9), LMG 21462 (S.S. w/o septum = 2.5; S.S. w/ septum = 2.2), and LMG 24506 (S.S. w/o septum = 2.4, S.S. w/ septum = 2.3). Target strains with a clinical origin were generally more sensitive than those with an environmental origin. These findings are in full agreement with previous data obtained for endophytes isolated from different medicinal plants [26].

The same test has been repeated using CoNS, *K. pneumoniae*, *P. aeruginosa*, and *S. aureus* stains as targets on standard Petri dishes. In this case (Tables 4 and 5), the I.S. for each *Arthrobacter* strain was less high than the one obtained in the test against Bcc. In particular, MS-3A20 (I.S. = 1.8); MS-3A3 (I.S. = 1.6) and MS-3A21 (I.S. = 1.5) showed a stronger inhibitory activity against CoNS strains, while the highest inhibition against *K. pneumoniae* was registered for the strain MS-3A4 (I.S. = 1.8). MS-2A14 (I.S. = 1.5) and MS-2A2 (I.S. = 1.4) were the most effective strains against *P. aeruginosa*, while MS-3A8 (I.S. = 1.3) scored the highest I.S. against *S. aureus*.

Overall, considering every I.S. for each *Arthrobacter* strain against the targets used in this study, MS-2A2, MS-3A4, MS-3A13, and MS-3A25 had the highest inhibition potential. As for the targets, *S. aureus* and *P. aeruginosa* were the least affected by the presence of the testers.

Another test was performed using Petri dishes with a physical septum to investigate the production of VOCs with an inhibitory activity against CoNS and *K. pneumoniae*, which were the most sensitive to the presence of the *Arthrobacter* strains. Data reported in Table 5 showed that the strains MS-2A2, MS-3A13, MS-3A17, MS-3A20, and MS-3A25 scored the highest I.S. against the targets *K. pneumoniae*.

**Table 3.**
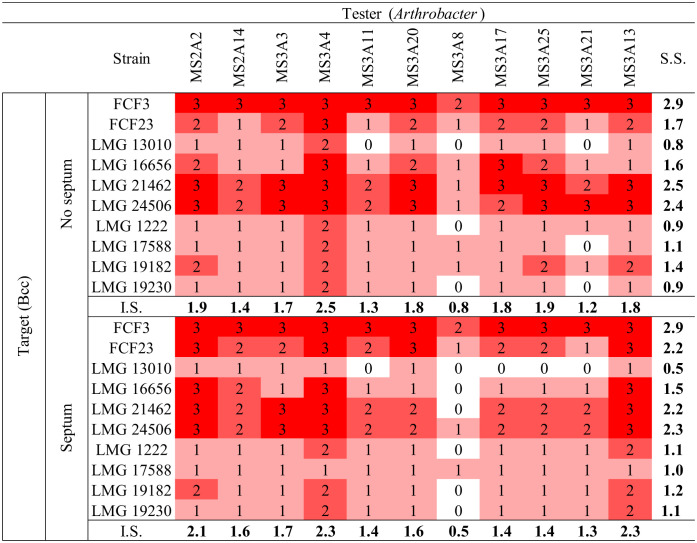
Cross-streaking test results: *Arthrobacter* against Bcc on Petri dishes with or w/o septum. The values were given according to how much the growth of the human pathogen had been negatively influenced by the tester strain. (0 = no inhibition; 1 = low inhibition; 2 = moderate inhibition; 3 = total inhibition).

Abbreviations: I.S., inhibition score; S.S., sensitivity score

**Table 4.**
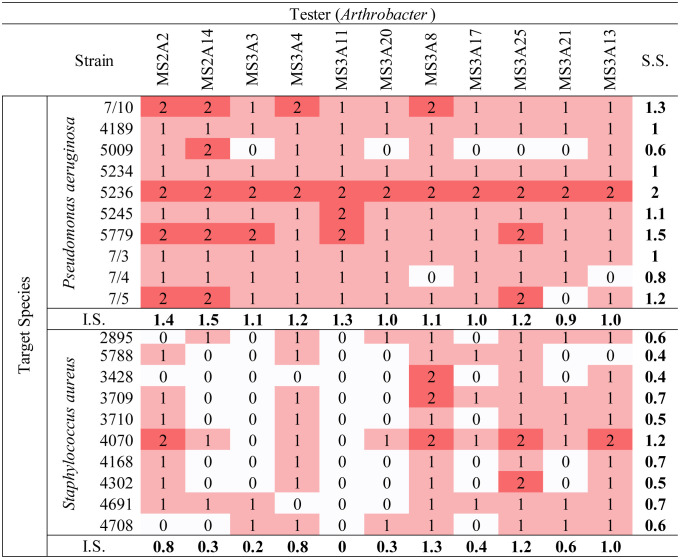
Cross-streaking test: *Arthrobacter* against *P. aeruginosa* and *S. aureus*. The values were given according to how much the growth of the human pathogen had been negatively influenced by the tester strain. (0= no inhibition; 1= low inhibition; 2= moderate inhibition; 3= total inhibition)

Abbreviations: I.S., inhibition score; S.S., sensitivity score

**Table 5.**
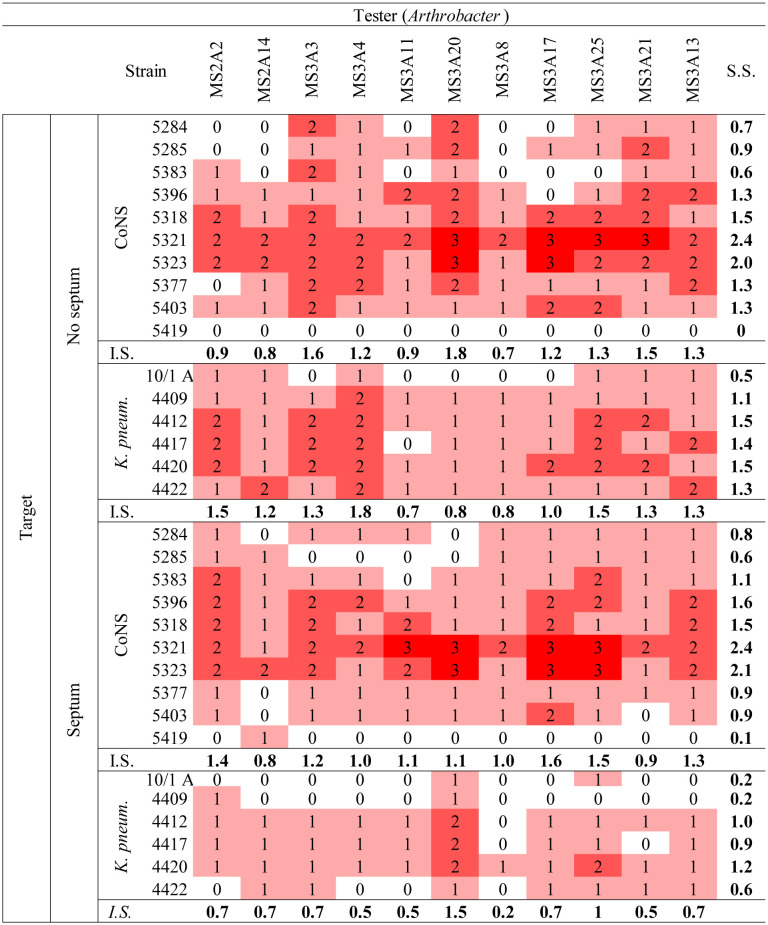
Cross-streaking test: *Arthrobacter* against CoNS and *K. pneumoniae* on Petri dishes with or w/o a physical septum. The values were given according to how much the growth of the human pathogen had been negatively influenced by the tester strain. (0 = no inhibition; 1 = low inhibition; 2 = moderate inhibition; 3 = total inhibition).

Abbreviations: I.S., inhibition score; S.S., sensibility score

### 3.5. Identification of VOCs by means of HS-GC/MS

**Figure 2.**
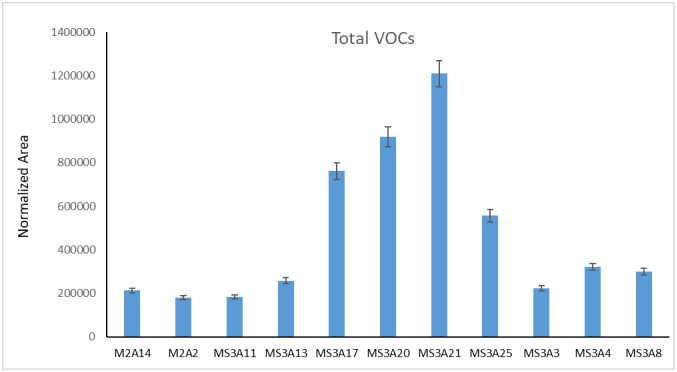
Total VOCs for each isolate calculated as total normalized area of the chromatogram peaks.

Each strain was analyzed by means of HS-GC/MS and the chemical profiles of identified VOCs are listed in Table 6. Results are expressed as the area of each peak normalized for biomass (Figure 2). Headspace-GC (HS-GC/MS) was chosen as it reduces sample manipulation, for its easy preparative steps and because it does not contemplate the use of solvents. A total of 16 distinct metabolites were detected, mainly belonging to the following structurally distinct classes: alcohols (1-butanol, 1-butanol-3-methyl,1-hexanol, 2-ethyl-, 2-propanol), ketones (2-butanone, 2-butanone, 3-methyl-, acetone), hemiterpene (isoprene), sulphurated compounds (bis(methylthio) methane, carbon disulfide, dimethyl sulfide, dimethyl disulfide, dimethyl trisulfide, ethanethioic acid S-methyl ester, metanthiol, thiophene) (Table 6).

Based on the relative peak area, the compound dimethyl disulfide was the most abundant VOC for MS-2A2, MS-2A14, MS-3A13, MS-3A4, MS-3A20, MS-3A17, MS-3A21, and MS-3A3 strains. Among sulfides, dimethylsulfide was also detected as an abundant metabolite produced by almost all strains.

**Figure 3.**
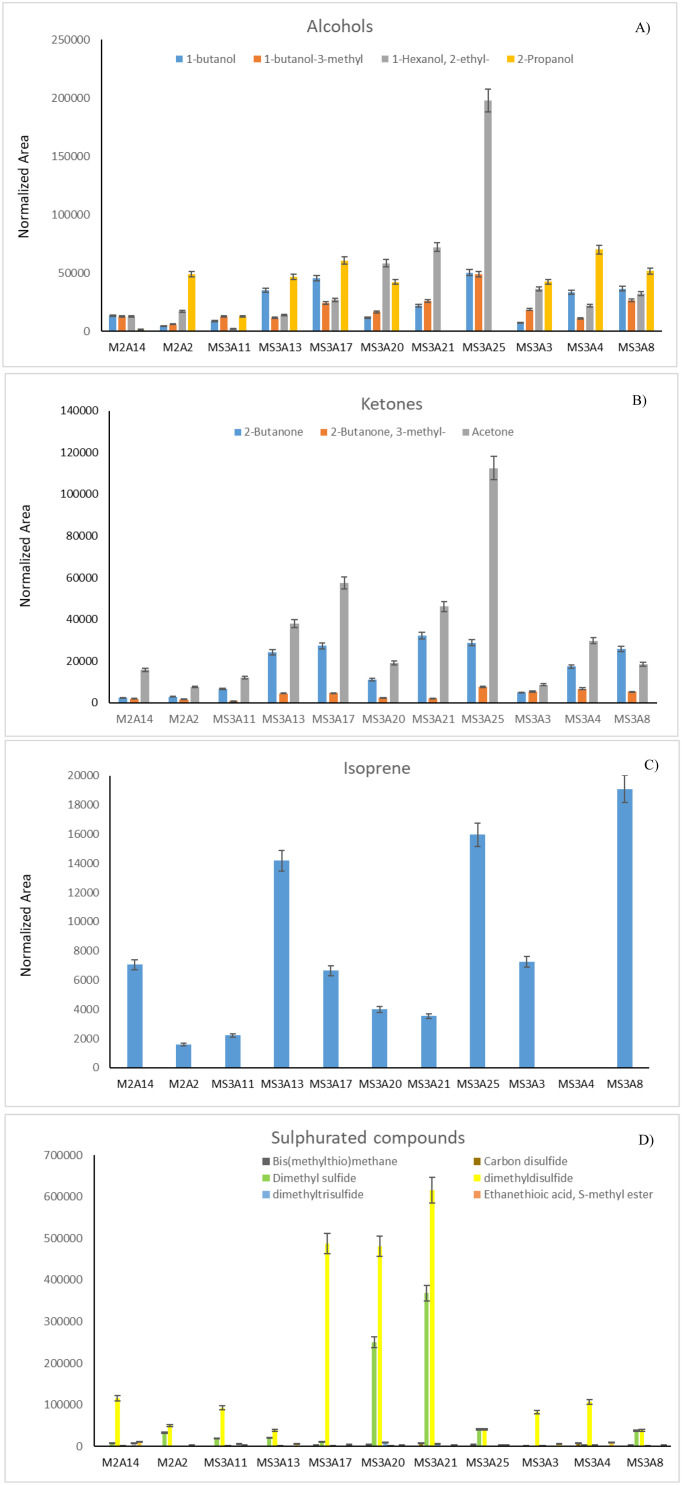
Distribution of identified (A) alcohols, (B) ketones, (C) isoprene, and (D) sulphurated compounds.

For strains MS-2A13 and MS-3A8 2-propanol was the most abundant compound. 1-hexanol-2-ethyl was the main component for MS-3A25. Distribution of various classes of metabolite among all strains are shown in Figure 3.

**Table 6.**
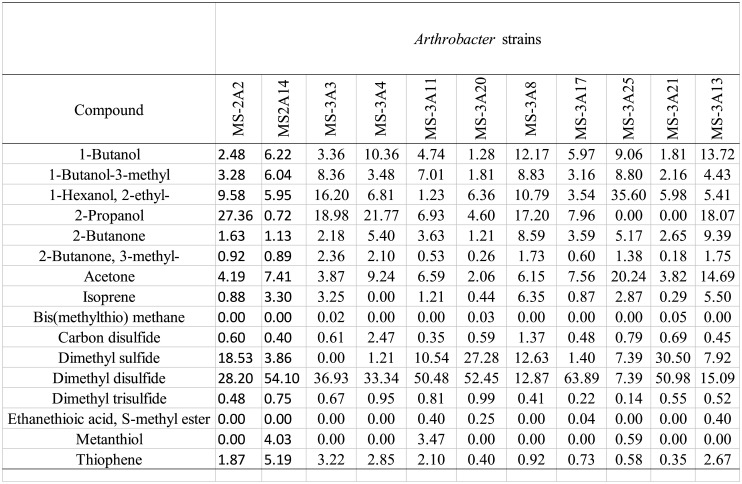
VOCs identified by HS-GC/MS and produced by *Arthrobacter* strains. Results are expressed as mean relative abundance percentages (as obtained by dividing the normalized area of each peak by the total area of the chromatogram peaks).

## 4. Discussion

Exploring the microbial diversity in extreme environments is a focal point of microbiology and environmental science research. Among these extraordinary habitats, the less studied Mongolian desert's arid expanses have captured the attention of scientists due to their harsh conditions and unique ecological niches [27].

This study focused on the molecular and phenotypic characterization of 11 *Arthrobacter* strains isolated from soil samples collected in 2 different oases in the Great Gobi A. The genus *Arthrobacter* stands out as a fascinating group of bacteria with a remarkable ability to adapt and thrive in diverse environmental conditions. *Arthrobacter* species have been discovered in various habitats, from soil and water to extreme environments like deserts and polar regions [25],[26]. These versatile microorganisms are particularly interesting due to their intriguing metabolic capabilities and potential applications in various fields, including bioremediation, agriculture, and biotechnology [28].

Concerning the molecular analyses, the RAPD analysis identified 11 different haplotypes, thus suggesting a high level of strain diversity, even if most bacteria were isolated from the same sample (sample 3A). The phylogenetic analysis based on 16S rDNA sequences revealed that the 11 *Arthrobacter* strains might belong to different species. *Arthrobacter* sp. MS-2A2, MS-2A14, MS-3A3, MS-3A4, MS-3A11, and MS-3A20 are close to *A. crystallopoietes*; MS-3A21 is close to *A. globiformis*; and MS-3A13 belongs to a sister group to the clade formed by *A. humicola, A. pascens*, and *A*. *oryzae*. MS-3A8, MS-3A17, and MS-3A25 form a clade separated from the other sequences. *A. crystallopoietes* possesses interesting characteristics that the phylogenetically close isolates may share, that is, the capability of resisting for prolonged periods under drought stress and starvation by converting only the 0.0005% of their cell carbon to CO_2_ within an hour [29]. This might suggest the adaptations of these isolates to the harsh conditions of the Gobi Desert, where water availability is scarce. Moreover, genes for the bioremediation of aromatic compounds, zinc, and sulphur from contaminated soils were found in the draft genome of *A. crystallopoietes* BAB-32 [30]. Other studies have shown its ability to degrade complex hydrocarbons, pesticides, and herbicides [31] as well as to reduce hexavalent chromium via a NADH-dependent chromate reductase [32]. Interestingly, these same strains (MS-2A2, MS-2A14, MS-3A3, MS-3A4, MS-3A11, and MS-3A20) show a higher resistance towards heavy metals. *A. globiformis* has been used to assess the quality of soil samples by analysing its dehydrogenase activity [33]. A thermostable histamine oxidase that is stable over a wide range of pH [34] has also been found in *A. crystallopoietes*, thus remarking the potential that the closely related isolates may synthesize thermostable proteins that could be exploited in different bioremediation contexts. *A. pascens* has been found in soil samples from the Himalayan cold desert and, apparently, presents a remarkable characteristic of maintaining metabolic activities at temperatures below 0 °C [35]. However, the *Arthrobacter* strain MS-3A13, which is phylogenetically close to *A. pascens*, does not show any evident psychrophilic characteristics.

Extreme environments present unique challenges for life and bacteria thriving in these conditions are thought to possess specialized adaptations. Plasmids can carry genes that provide these adaptations, such as resistance to extreme temperatures, high salinity, or toxic compounds. They can carry also antibiotic resistance genes that might harbor genetic code for enzymes suitable for industrial processes, bioremediation, or the production of valuable compounds [36]. The presence of plasmids in the 11 *Arthrobacter* strains was evaluated but data obtained indicated the absence of plasmids. However, this does not exclude the presence of larger plasmids that may contain genes for antibiotic resistance or virulence factors that may be clinically relevant.

Extreme environments may host unique microbial communities that could harbour new antibiotic resistance variants [37]. Monitoring the presence and evolution of antibiotic resistance in these bacteria is essential for global antibiotic resistance surveillance. Moreover, discovering antibiotic resistance in bacteria isolated from extreme environments can have direct implications for public health, in that it may signal that such resistances are spreading more widely in the environment, with the potential to compromise the effectiveness of antibiotics in treating human infections [38]. Each *Arthrobacter* strain resisted to high concentrations of kanamycin and streptomycin while being less tolerant towards chloramphenicol, ciprofloxacin, rifampicin, and tetracycline. Interestingly, strains MS-3A25, MS-3A8, and MS-3A17, which are phylogenetically closer to each other than to any other type-strain considered, exhibited a higher resistance to streptomycin and ciprofloxacin. These resistance mechanisms could have evolved as a response to various environmental stressors. It was observed that the abundance of antibiotic resistance genetic features in the bacterial communities of the desert environment significantly increased when in the proximity of an oasis, due to human activity [10].

The *Arthrobacter* strains were capable of growing in the presence of moderate concentrations of KH_2_AsO_4_ and NaAsO_2_, while low concentrations of CuCl_2_, ZnSO_4_, Cd(NO_3_), and NiCl_2_ incapacitate their growth. As stated above, the isolates that are closely related to *A. crystallopoietes* are the ones with a higher resistance to heavy metals. Moreover, they tolerated concentrations below 2% of NaCl, while concentrations higher than 4% lead to total growth inhibition for each one of the isolates.

A comparison of the MIC of different antibiotics and heavy metals between *Arthrobacter* strains isolated from Gobi Desert and associated with *Origanum heracleoticum* L. showed that strains belonging to the same genus do not share the same resistance profiles, even though many of the analyzed strains are phylogenetically close (Supplementary Figure S5), The Mongolia Soil *Arthrobacter* strains split form the *O. heracleoticum* associated strains in the PCA plot (Supplementary Figure S6), the resistance to kanamycin, streptomycin, and arsenate (As(V)) being the most influential differences between the two groups. This might be related to the differences in the characteristics of the environment they inhabit.

Of utmost importance in the context of this work are the data concerning the ability of the 11 *Arthrobacter* strains to interfere with the growth of bacterial human pathogens belonging to different species. The increasing emergence of antibiotic resistant pathogens, as opposed to the slow progress in the field of chemical synthesis of antimicrobial compounds, demands researchers to seek novel antibiotics [39]. Antibiotics produced by desert microorganisms may have unique properties or novel chemical structures and can be of interest for biotechnological applications, including the development of new antibiotics or other pharmaceuticals. Cross-streaking tests against human pathogens were conducted to test the *Arthrobacter* strains' ability to produce diffusible molecules and VOCs with antimicrobial potential. The pathogens towards which these isolates were most active were the Bcc strains of clinical origin. Similarly, the VOCs produced by *Arthrobacter* strain OVS8, isolated from the stem inner tissues of the aromatic plant *Origanum vulgare* L., strongly inhibited the growth of Bcc strains [23]. The antagonistic activity towards *S. aureus*, CoNS, *P. aeruginosa*, and *K. pneumoniae* strains was lower, likely due to the multiple antibiotic resistances possessed by such target strains.

The volatilome of each *Arthrobacter* strain was further investigated by HS-GC/MS analysis. A total of 16 distinct metabolites were detected, mainly belonging to structurally distinct classes. Among sulfides, dimethylsulfide was also detected as an abundant metabolite produced by almost all strains. It was demonstrated that dimethyl disulfide causes significant inhibition of different pathogens such as *Rhizoctonia solani* and *Pythium ultimum*, or many Gram-negative and Gram-positive bacteria [40]. The only correspondence found between HS-GC/MS data and the cross-streaking results is the higher production of thiophene, ethanethioic acid S-methyl ester, butanol, propanol, and acetone by MS-3A13. This same strain always scored one of the highest I.S. against Bcc, CoNS, and *K. pneumoniae* strains when using plates with a physical septum. These results are in accordance with other studies [23], underlying the importance of understanding how the production of VOCs is related to the antimicrobial capacity of the *Arthrobacter* genus against human pathogens and especially against Bcc. The function of VOCs is yet to be clearly understood from an ecological perspective [23], however they might have a role in the regulation of atmospheric chemistry, soil transformation and biotic interaction in soil. Even if studies have demonstrated that VOC production is constitutive rather than influenced by the presence of human pathogens (Bcc) [41], it is likely that they could constitute a means for intra and inter-specific communication [42] as their production is influenced by the environmental characteristics in which bacteria are found [23].

Overall, data obtained in this work suggest that bacteria inhabiting the extreme environment represented by the Great Gobi A Strictly Protected Area might represent a valuable source of new natural antimicrobial compounds able to inhibit the growth of very important multi-drug resistance human pathogens.

## Use of AI tools declaration

The authors declare they have not used Artificial Intelligence (AI) tools in the creation of this article.


