# Supplementary material for: Antibacterial activity of Arthrobacter strains isolated from Great Gobi A Strictly Protected Area, Mongolia
Source: AIMS Microbiol. 2024 Feb 28;10(1):161–86. doi: 10.3934/microbiol.2024009 (PMC10955175; doi:10.3934/microbiol.2024009)
Supplement: Supplementary file 1 [file microbiol-10-01-009-s001.pdf]

Microbiology

AIMS Microbiology, 10(1): 161–186.
DOI: 10.3934/microbiol.2024009
Received: 27 October 2023
Revised: 01 February 2024
Accepted: 22 February 2024
Published: 28 February 2024

http://www.aimspress.com/journal/microbiology

Research article

Antibacterial activity of *Arthrobacter* strains isolated from Great Gobi A Strictly Protected Area, Mongolia

Alberto Bernacchi[1], Giulia Semenzato[1], Manuel di Mascolo[1], Sara Amata[2], Angela Bechini[3], Fabiola Berti[3], Carmela Calonico[3], Valentina Catania[4], Giovanni Emiliani[5], Antonia Esposito[6], Claudia Greco[7], Stefano Mocali[6], Nadia Mucci[7], Anna Padula[7], Antonio Palumbo Piccionello[2], Battogtokh Nasanbat[9], Gantulga Davaakhuu[9], Munkhtsetseg Bazarragchaa[10], Francesco Riga[11], Claudio Augugliaro[12], Anna Maria Puglia[8], Marco Zaccaroni[1] and Fani Renato[1,*]

[1] Department of Biology, University of Florence, Via Madonna del Piano 6, Sesto Fiorentino, 50019 Florence, Italy
[2] Department of Biological, Chemical and Pharmaceutical Sciences and Technologies-STEBICEF, University of Palermo, Viale delle Scienze Ed.17, 90128, Palermo, Italy
[3] Department of Health Sciences, University of Florence, viale G.B. Morgagni, 48, 50134 Firenze, Italy
[4] Department of Earth and Sea Science (DiSTeM), University of Palermo, Viale delle Scienze Blg. 16, Palermo, 90128, Italy
[5] Institute for Sustainable Plant Protection (IPSP)—National Research Council (CNR), Via Madonna del Piano 10, Sesto Fiorentino 50019 Florence, Italy
[6] Council for Agricultural and Economics Research (CREA) – Agriculture and Environment, Via di Lanciola 12/A, Cascine del Riccio, 50125, Florence, Italy
[7] Unit for Conservation Genetics (BIO-CGE), Institute for Environmental Protection and Research, via Ca' Fornacetta, 9, 40064 Ozzano dell'Emilia Bologna, Italy
[8] Consorzio Italbiotec, Piazza della Trivulziana 4/a 20126 Milano
[9] Institute of Biology, Mongolian Academy of Sciences, Peace Avenue-54B, Bayanzurkh District, Ulaanbaatar-13330, Mongolia
[10] Mongolian National University of Medical Sciences, S.Zorig street, Ulaanbaatar-14210, Mongolia
[11] Italian Institute for Envioronmental Protection and Research, via Vitalino Brancati, 48, 00144, Roma, Italy
[12] Wildlife Initiative, Bayangol, 6th Khoroo, Micro District 10, Ulaanbaatar, 210349, Mongolia

* **Correspondence:** Email: renato.fani@unifi.it; Tel: +390554574742.

Supplementary

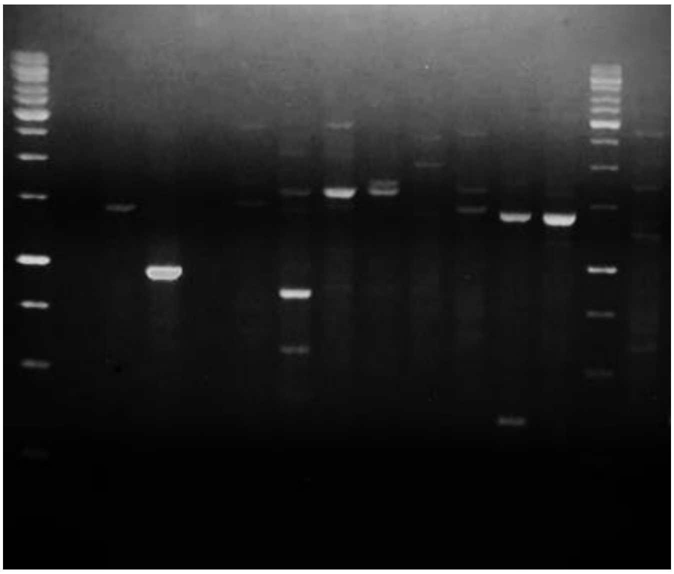

Figure S1. RAPD visualization on agarose gel. The amplicons for each strain are shown in the following order: MS2A2; MS3A4; MS2A14; MS3A8; MS3A11; MS3A13; MS3A6; MS3A17; MS3A25; MS3A3; MS3A7; MS3A20; MS3A21.

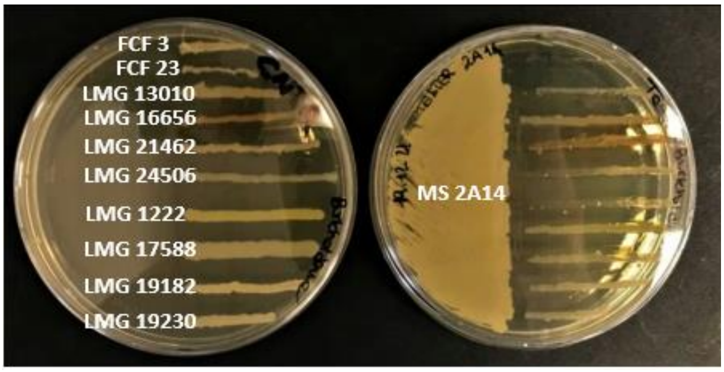

Figure S2. Cross Streaking test on Petri dishes without physical septum. Tester: *Arthrobacter* MS2A14; Targets from top to bottom: *Burkholderia cepacia* FCF3, *B. cenocepacia* FCF23, *B. cenocepacia* LMG 13010, *B. cenocepacia* LMG 16656, *B. cenocepacia* LMG 21462, *B. cenocepacia* LMG 24506, *B. cepacia* LMG 1222, *B. multivorans* LMG 17588, *B. ambifaria* LMG 19182, *B. cenocepacia* LMG 19230.

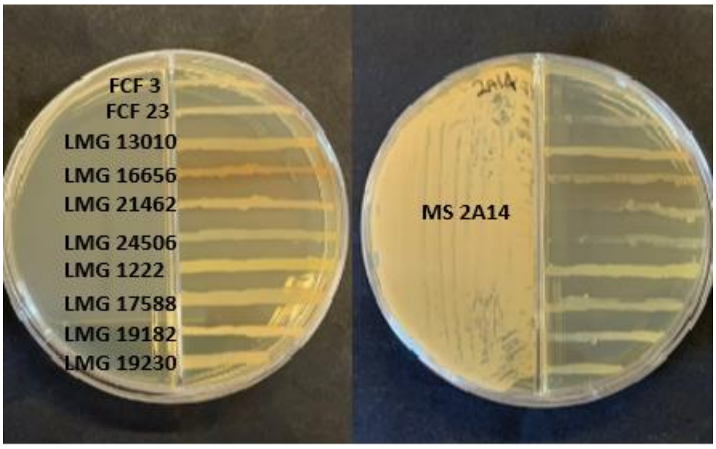

Figure S3. Cross Streaking test on Petri dishes with physical septum. Tester: *Arthrobacter* MS2A14; Targets from top to bottom: *Burkholderia cepacia* FCF3, *B. cenocepacia* FCF23, *B. cenocepacia* LMG 13010, *B. cenocepacia* LMG 16656, *B. cenocepacia* LMG 21462, *B. cenocepacia* LMG 24506, *B. cepacia* LMG 1222, *B. multivorans* LMG 17588, *B. ambifaria* LMG 19182, *B. cenocepacia* LMG 19230.

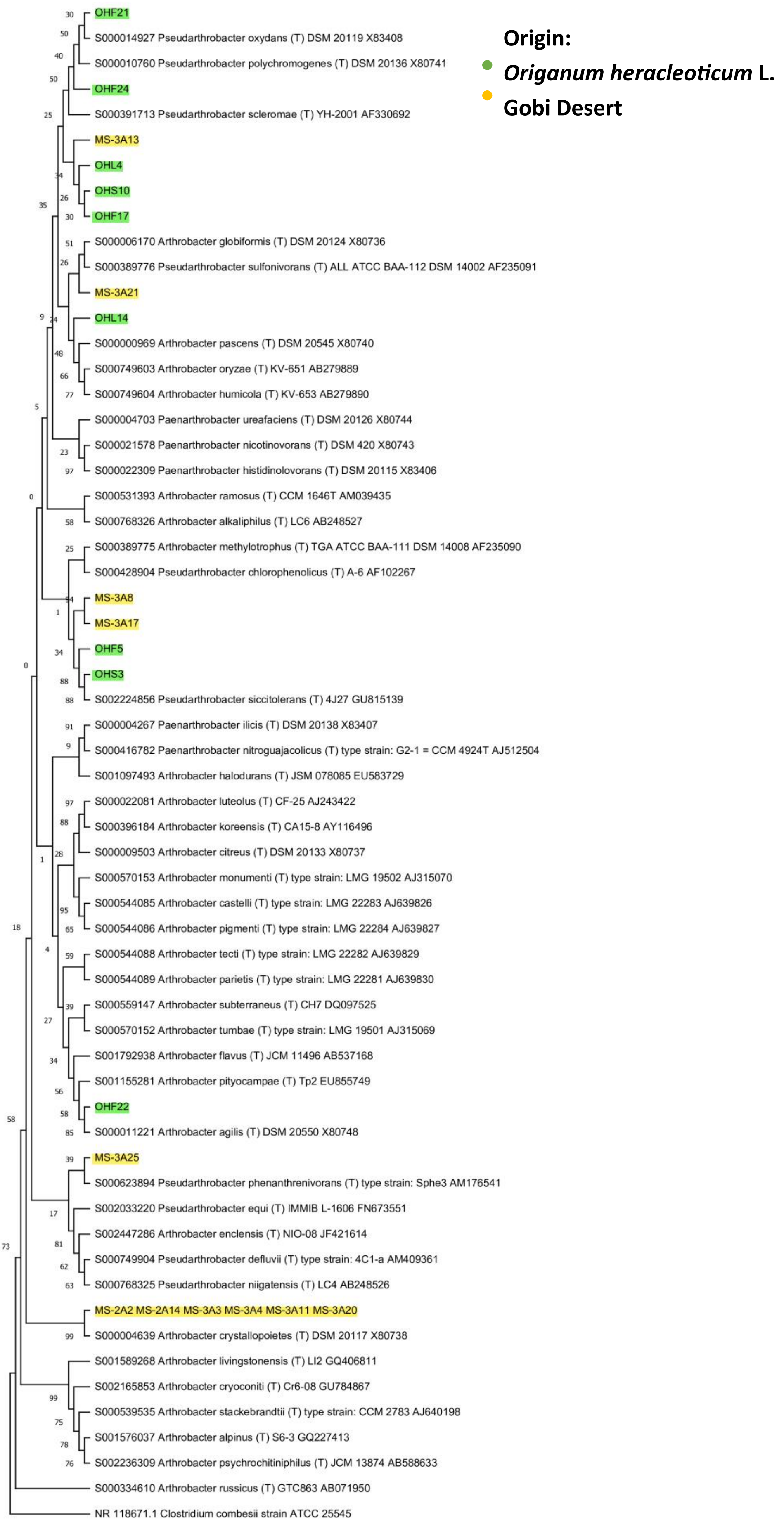
Origin:
Origanum heracleoticum L.
Gobi Desert
OHF21
S000014927 Pseudarthrobacter oxydans (T) DSM 20119 X83408
S000010760 Pseudarthrobacter polychromogenes (T) DSM 20136 X80741
OHF24
S000391713 Pseudarthrobacter scleromae (T) YH-2001 AF330692
MS-3A13
OHL4
OHS10
OHF17
S000006170 Arthrobacter globiformis (T) DSM 20124 X80736
S000389776 Pseudarthrobacter sulfonivorans (T) ALL ATCC BAA-112 DSM 14002 AF235091
MS-3A21
OHL14
S000000969 Arthrobacter pascens (T) DSM 20545 X80740
S000749603 Arthrobacter oryzae (T) KV-651 AB279889
S000749604 Arthrobacter humicola (T) KV-653 AB279890
S000004703 Paenarthrobacter ureafaciens (T) DSM 20126 X80744
S000021578 Paenarthrobacter nicotinovorans (T) DSM 420 X80743
S000022309 Paenarthrobacter histidinolovorans (T) DSM 20115 X83406
S000531393 Arthrobacter ramosus (T) CCM 1646T AM039435
S000768326 Arthrobacter alkaliphilus (T) LC6 AB248527
S000389775 Arthrobacter methylotrophus (T) TGA ATCC BAA-111 DSM 14008 AF235090
S000428904 Pseudarthrobacter chlorophenolicus (T) A-6 AF102267
MS-3A8
MS-3A17
OHF5
OHS3
S002224856 Pseudarthrobacter siccitolerans (T) 4J27 GU815139
S000004267 Paenarthrobacter ilicis (T) DSM 20138 X83407
S000416782 Paenarthrobacter nitroguajacolicus (T) type strain: G2-1 = CCM 4924T AJ512504
S001097493 Arthrobacter halodurans (T) JSM 078085 EU583729
S000022081 Arthrobacter luteolus (T) CF-25 AJ243422
S000396184 Arthrobacter koreensis (T) CA15-8 AY116496
S000009503 Arthrobacter citreus (T) DSM 20133 X80737
S000570153 Arthrobacter monumenti (T) type strain: LMG 19502 AJ315070
S000544085 Arthrobacter castelli (T) type strain: LMG 22283 AJ639826
S000544086 Arthrobacter pigmenti (T) type strain: LMG 22284 AJ639827
S000544088 Arthrobacter tecti (T) type strain: LMG 22282 AJ639829
S000544089 Arthrobacter parietis (T) type strain: LMG 22281 AJ639830
S000559147 Arthrobacter subterraneus (T) CH7 DQ097525
S000570152 Arthrobacter tumbae (T) type strain: LMG 19501 AJ315069
S001792938 Arthrobacter flavus (T) JCM 11496 AB537168
S001155281 Arthrobacter pityocampae (T) Tp2 EU855749
OHF22
S000011221 Arthrobacter agilis (T) DSM 20550 X80748
MS-3A25
S000623894 Pseudarthrobacter phenanthrenivorans (T) type strain: Sphe3 AM176541
S002033220 Pseudarthrobacter equi (T) IMMIB L-1606 FN673551
S002447286 Arthrobacter enclensis (T) NIO-08 JF421614
S000749904 Pseudarthrobacter defluvii (T) type strain: 4C1-a AM409361
S000768325 Pseudarthrobacter niigatensis (T) LC4 AB248526
MS-2A2 MS-2A14 MS-3A3 MS-3A4 MS-3A11 MS-3A20
S000004639 Arthrobacter crystallopoietes (T) DSM 20117 X80738
S001589268 Arthrobacter livingstonensis (T) LI2 GQ406811
S002165853 Arthrobacter cryoconiti (T) Cr6-08 GU784867
S000539535 Arthrobacter stackebrandtii (T) type strain: CCM 2783 AJ640198
S001576037 Arthrobacter alpinus (T) S6-3 GQ227413
S002236309 Arthrobacter psychrochitiniphilus (T) JCM 13874 AB588633
S000334610 Arthrobacter russicus (T) GTC863 AB071950
NR 118671.1 Clostridium combesii strain ATCC 25545

Figure S4 Phylogenetic tree base on 16S rRNA coding genes of *Arthrobacter* strains isolated from Gobi Desert soil (MS-) or associated to *Origanum heracleoticum* L. (OH-). The *Clostridium combesii* orthologous sequence was used as an outgroup.

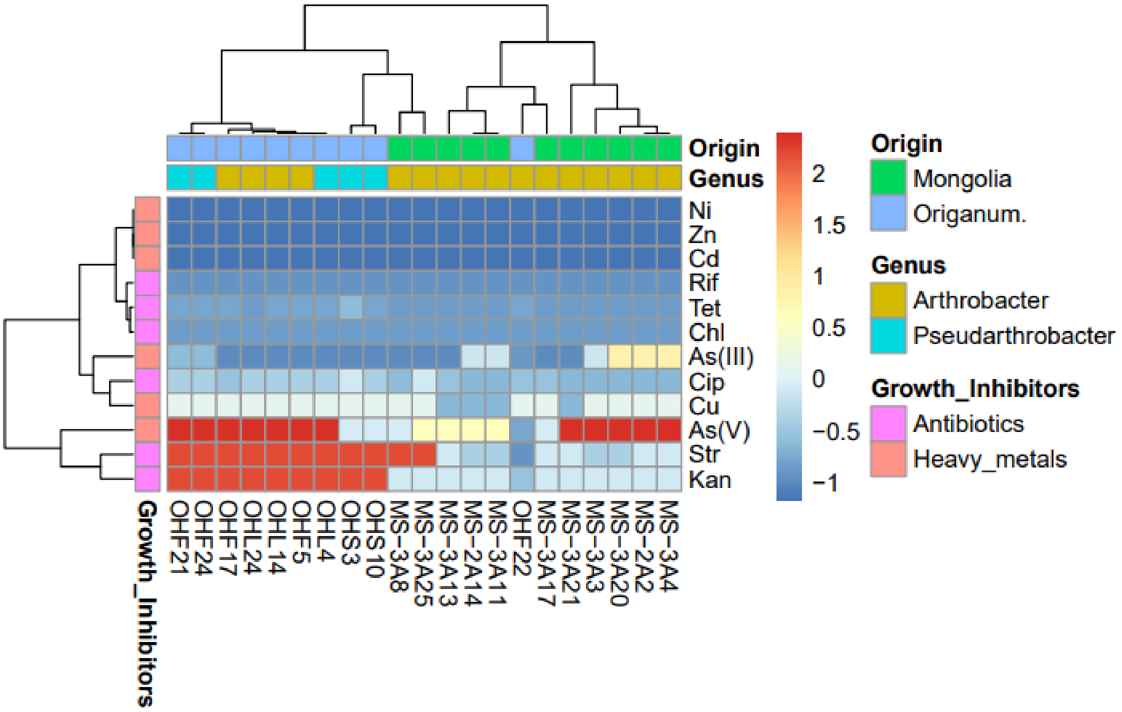

Figure S5. Heatmap of the MIC of different antibiotics and heavy metals for *Arthrobacter* strains isolated from Gobi Desert soil (MS-) or associated to *O. heracleoticum* L. (OH-). The values were obtained by normalizing the MIC values.

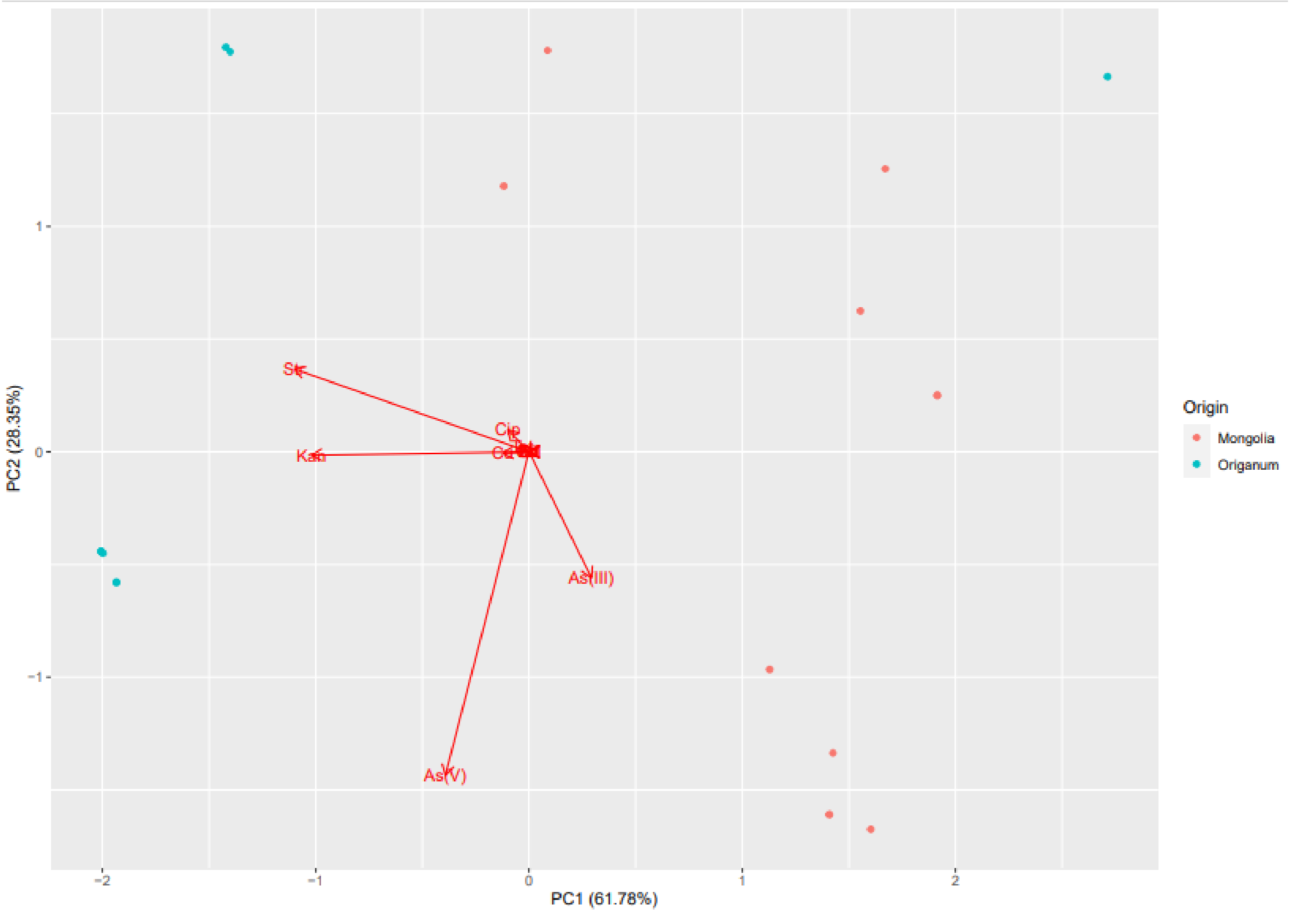

Figure S6. PCA plot showing the differences between the *Arthrobacter* strains isolated from Gobi Desert soil and those associated with *O. heracleoticum* L.

AIMS Press

© 2024 the Author(s), licensee AIMS Press. This is an open access article distributed under the terms of the Creative Commons Attribution License (http://creativecommons.org/licenses/by/4.0)
